# Characterization of the *Zoarces viviparus *liver transcriptome using massively parallel pyrosequencing

**DOI:** 10.1186/1471-2164-10-345

**Published:** 2009-07-31

**Authors:** Erik Kristiansson, Noomi Asker, Lars Förlin, DG Joakim Larsson

**Affiliations:** 1 Department of Zoology, University of Gothenburg, Box 463, SE-405 30, Göteborg, Sweden; 2 Department of Neuroscience and Physiology, the Sahlgrenska Academy at the University of Gothenburg, Box 434, SE-405 30 Göteborg, Sweden

## Abstract

**Background:**

The teleost *Zoarces viviparus *(eelpout) lives along the coasts of Northern Europe and has long been an established model organism for marine ecology and environmental monitoring. The scarce information about this species genome has however restrained the use of efficient molecular-level assays, such as gene expression microarrays.

**Results:**

In the present study we present the first comprehensive characterization of the *Zoarces viviparus *liver transcriptome. From 400,000 reads generated by massively parallel pyrosequencing, more than 50,000 pieces of putative transcripts were assembled, annotated and functionally classified. The data was estimated to cover roughly 40% of the total transcriptome and homologues for about half of the genes of *Gasterosteus aculeatus *(stickleback) were identified. The sequence data was consequently used to design an oligonucleotide microarray for large-scale gene expression analysis.

**Conclusion:**

Our results show that one run using a Genome Sequencer FLX from 454 Life Science/Roche generates enough genomic information for adequate *de novo *assembly of a large number of genes in a higher vertebrate. The generated sequence data, including the validated microarray probes, are publicly available to promote genome-wide research in *Zoarces viviparus*.

## Background

*Zoarces viviparus*, or common eelpout, lives in the shallow waters along the coasts in Northern Europe [1]. In contrast to many other fish species living in these regions, the eelpout has a number of distinct characteristics which makes this fish particularly interesting for ecological and ecotoxicological studies. The relatively stationary behavior makes it possible to correlate physiological changes to the local exposure situation in the area where the fish were sampled [2]. This makes the eelpout a good candidate for environmental monitoring since it is possible to associate adverse effect to specific pollutants or pollution sources. Another beneficial characteristic is the internal fertilization where the females, after several months of pregnancy, give birth to relatively well-developed young. The possibility to link the mother with her offspring makes the eelpout suited for investigations of reproductive success, both in general and under the exposure of pollutants [3].

The value of the eelpout as an important species for coastal environmental monitoring in the field has been demonstrated in a number of studies. Exposure to effluent from Scandinavia's largest pulp mill results in skewed sex ratio of eelpout embryos [4,5]. Excessive proportions of malformed and/or dead eelpout embryos have revealed the impact of environmental pollution at several locations along the coast of the Baltic Sea [6,7]. Climate-induced temperature shifts have also been linked to reduced growth of eelpout [8]. As a species high on the food web of the marine ecosystems, the eelpout is known to bioaccumulate toxic substances such as heavy metals [9] and the polycyclic aromatic hydrocarbons [10]. *Z. viviparus *is therefore a prime target for the German Environmental Specimen Bank (ESB), who monitors and identifies local and regional trends of the aquatic ecosystems along the German coasts.

Genomic-based techniques are today important tools for investigating molecular level effects caused by exposure of toxicants and other substances. Large-scale measurements of gene expression using DNA microarrays have become particular popular since they provide both quantitative data for individual biomarkers [11,12] as well as a genome-wide snapshot of the current state of the cells [13,14]. As a consequence, groups of genes and entire pathways can be associated with different types of toxicity [15], which leads to both improved ability to detect exposures and an increased knowledge of the mechanisms of action and subsequent adverse effects [12,16,17]. However, microarray-based gene expression measurements require a substantial amount of knowledge about the targeted species' transcriptome, and these valuable tools are therefore unavailable for many ecologically important species.

During the last years there has been a remarkable progress in DNA sequencing technology with several novel solutions on the market [18-20]. The performance has increased multiple orders of magnitude and millions of bases can today be sequenced in a matter of hours [21]. Within the fields of ecology and ecotoxicology, this development is particularly welcome due to the large number of model organisms, of which few have a well-characterized genome. The new fast and cost-efficient methods for DNA sequencing hold therefore great promise to change the current situation and pave the way for modern genomic tools such as gene expression microarrays [22] and genome-wide association population studies [23,24]. For transcriptome sequencing, the superior performance of high-throughput techniques have already been demonstrated in several studies, including the plant *Medicago truncatula *[25], the butterfly *Melitaea cinxia *[26], the tree *Eucalyptus grandis *[27] and the freshwater fish *Micropterus salmoides *[28].

The novel sequencing techniques also comes with a number of weaknesses where the limited sequence length is most pronounced [29]. Among the currently available techniques, the FLX pyrosequencer from 454 Life Science/Roche generates the longest reads, currently 250–400 bases depending on the protocol, and is therefore most well-suited for *de novo *transcriptome sequencing [30]. Even though hundreds of thousands reads are generated simultaneous, the limited length will severely restrain the assembly of full length transcripts [27].

In this study we present the first comprehensive characterization of the *Z. viviparus *liver transcriptome using massively parallel pyrosequencing. Based on data corresponding to one single run on the FLX Gene Sequencer from 454 Life Science, almost 100 million bases were assembled into ~53,000 pieces of putative transcripts and the majority of these have been annotated and functionally classified. Furthermore, an oligonucleotide microarray for large-scale gene expression measurement in *Z. viviparus *has been designed. Our results, including the sequence data and the validated microarray probes are publicly available and will provide means for future genome-wide research in *Z. viviparus*.

## Results

### Sequence analysis and quality assessment

The polymer chain reaction (PCR) amplification adapters used in the sequencing process were first identified and removed; the start tag was found for 99% of the reads while the end tag only was found for 42%, indicating that the majority of the reads have reached their maximum length and less than half stopped prematurely. A substantial part of the genome of higher eukaryotes consists of stretches with low-complexity such as the short and long interspaced repetitive elements (SINEs and LINEs) and other transposons [31] and these regions makes gene assembly treacherous since reads can be conjoined because they share a similar repetitive region. This is also true for the poly-A-tail, the A/T rich region that is attached to the 3' end of the mRNA molecule after transcription. The entire dataset was therefore screened for low-complexity regions and poly-A tails. As expected, the number of identified poly-A tails was low (<1% of the reads) compared to traditional cloned EST library due to the uniform distribution of read positions exhibited by parallel pyrosequencing [32]. In total, 1.5 million bases were classified as low-complex, and were either substituted for ambiguity bases or removed whether their location was intermediate or in the ends.

### Assembly of transcripts

**Figure 1:**
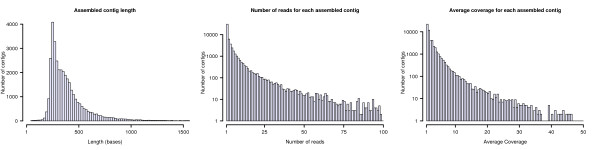
**A summary of the sequencing and assembly of the *Z. viviparus *transcriptome**. The three histograms show the distribution of the length, the number of reads and the average coverage of the assembled contigs.

To assemble the reads into transcripts, we applied the TGI Clustering Tools (TGICT), which is a set of utilities originally developed for assembly of data from cloned EST libraries. The assembly resulted in 36,110 contiguous sequences (contigs), while 17,347 reads could not be matched against any other (singlets), resulting in 53,447 putative eelpout transcripts consisting of more than 18 million bases in total (Table 1). The average length of the contigs was ~400 base pairs, with more than 7,000 contigs with a length over 500 base pairs (Figure 1a).

**Table 1:** A summary of the assembled transcripts

	**Contigs**	**Singlets**	**Total**
Number of sequences	36,110	17,347	53,457
Number of bases	14,250,156	4,050,061	18,300,217
Average length (bases)	395	233	342
Max length (bases)	3,401	608	3,401
GC-content	46.8%	43.5%	46.1%
Average coverage	3.46	1	2.67
Found in Uniprot^a^	73.4%	69.8%	72.2%
Found in at least one of the five available fish genomes^a, b^	89.2%	87.3%	88.6%

^a ^Using a BLAST E-value cut-off of 10^-5^

^b ^*Gasterosteus aculeatus*, *Tetraodon nigroviridis*, *Takifugu rubripes, Oryzias latipes *and *Danio rerio*.

The number of reads per contig ranged up to ~2,000 with an average of eight reads (Figure 1b) and, even though the cDNA library was normalized prior to sequencing, the distribution was highly asymmetric and right-tailed. The coverage, i.e. the average number of supporting reads per base pair, was estimated to 3.5 times (Figure 1c). With the low error rate of massively parallel pyrosequencing in mind [33], these results suggest that the assembly should be of high quality with very few technical errors.

### Annotation

The assembled transcripts were annotated against Swiss-Prot/UniProtKB [34], a manually curated database containing ~350,000 known protein from more than 10,000 different species. Using a modest E-value cut-off of 10^-5^, 73% of the contigs and 70% of the singlets had a homologous hit, resulting in 38,620 annotated eelpout transcripts. Analogously, 89% of the contigs and 87% of the singles had a hit in at least one of the five available fish genomes.

A closer examination of the annotations revealed several genes that are of particular interest for environmental monitoring and ecological research (Table 2). Several proteins within the cytochrome P450 protein superfamily catalyzes biotransformation of many xenobiotics and therefore contains a number of pivotal biomarkers for a large number of different compounds. Among the sequenced *Z. viviparus *transcripts, 45 different cytochrome P450 variants from 11 different families could be identified (e.g. CYP1A and CYP3A). Other interesting classes of proteins, such as heat shock proteins (e.g. HSP70, HSP90 alpha and beta) and genes related to oxidative stress (e.g. superoxide dismutase and glutathione peroxidase) were also present, as well as several other common biomarkers, such as vitellogenin, the zona pellucida and metalothionein proteins.

**Table 2:** Examples of biomarker genes of ecotoxicological interest

	Parallel pyrosequencing		GenBank	
**Gene**	Accession	Length (bases)^a^	Accession	Length (bases)

Vitellogenin	ZOVI0010766	1,826	AJ416326	1,229
Zona Pelucida 2	ZOVI0014264	1,100	-	-
Zona Pelucida 3	ZOVI0034606	989	-	-
Estrogen receptor	ZOVI0044876	852	AY223902	3,256
Metallothionein	ZOVI0049137	363	X97270	312
Heat-shock protein 70	ZOVI0038668	1,460	-	-
Heat-shock protein 90	ZOVI0020982	938	-	-
Cytochrome P450 1A	ZOVI0005392	1,652	-	-
Superoxide dismutase	ZOVI0007529	747	-	-
Glutathione peroxidase	ZOVI0037346	1,208	-	-

^a^Length of longest contiguous fragment

### Comparative genomics

Among the five fish species with sequence genome, *Gasterosteus aculeatus *(stickleback) is evolutionarily closest to *Z. viviparus *and the genome encompasses 20,000 protein coding genes which have a total size of 45 million base pairs. Assuming similar numbers in eelpout, the assembled transcripts would cover roughly 40% of the transcriptome and each gene would on average be represented by more than two contigs or singlets. The GC-content differed between the two species, with *G. aculeatus *at 55% while *Z. viviparus *had 48% for the contigs but only 43% for the singlets.

**Figure 2:**
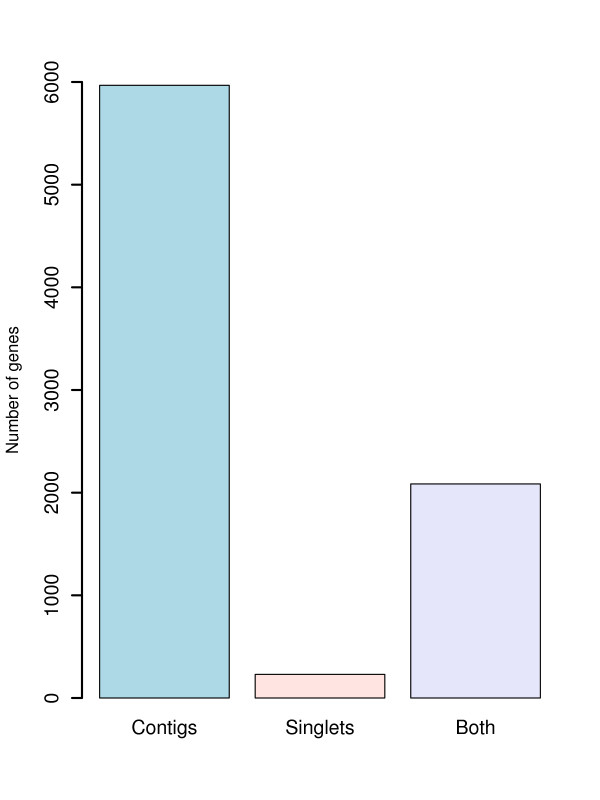
**The number of genes in *Gasterosteus aculeatus *(stickleback), the evolutionary closest species with a sequenced genome, matched by at least one *Z. viviparus *transcript**. The numbers were divided into genes only matched by contigs, genes only matched by singlets and genes matched by both.

The 53,447 putative *Z. viviparus *transcripts matched 8,283 unique genes in *G. aculeatus *and among these, 72% corresponded to hits from contigs and 3% from singlets while more than 25% had hits from both contigs and singlets (Figure 2). Hence, most singlet are therefore from genes already represented by a least one contig and very few genes are represented only as singlets. Even though the number of unique genes decreased slightly, the conclusions were consistent for the other four fish species with sequenced genome (*Tetraodon nigroviridis*, *Takifugu rubripes*, *Oryzias latipes*, *Danio rerio*). Not surprisingly, the number of human genes with a match from *Z. viviparus *transcripts was lower than all the fish but slightly higher than the frog (*Xenopus tropicalis*) (see Additional file 1).

**Figure 3:**
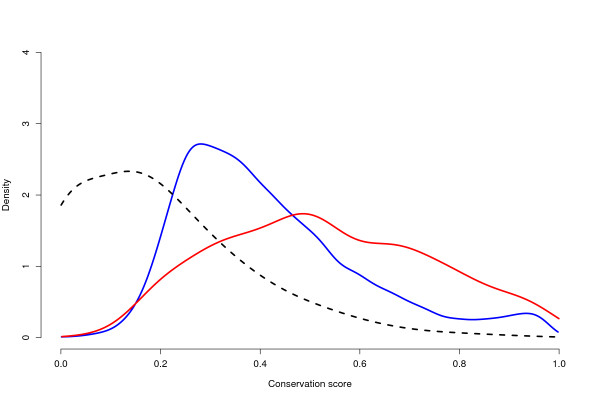
**Densities for the evolutionary conservation of 18,717 *Z. viviparus *transcripts found in at least three of the five available fish genomes**. Transcript aligning outside (red line) are significantly more conserved than those aligning inside (blue line) annotated regions (p < 10^-16^, Wilcoxon-Mann-Whitney rank sum test). For comparison, a null distribution was generated by calculating the conservation score for random alignments, with similar length as the transcripts aligning outside annotated regions (dashed line).

To investigate the presence of novel previously undescribed genes, all contigs and singlets were aligned against the five available fish genomes. In total 18,717 of the transcripts could be found in at least three of the five fish species with sequenced genome, and among those, 621 (3.3%) aligned in regions without previous annotation. Not surprisingly, only 367 (59%) of these could not be annotated against UniProt, which is lower than the transcripts aligning in annotated regions (88%, p < 10^-16^, Fisher's exact test). The transcripts in the regions without annotation had a slightly higher coverage (2.23 to 2.02 reads/base, p = 0.037, Mann-Whitney-Wilcoxon rank sum test) and a similar number of reads per transcript (the median number of reads is 3 for both groups). More interestingly, these unknown transcripts aligned in regions with a higher evolutionary conservation (Figure 3) compared to transcripts aligning in annotated regions (median evolutionary conservation score 0.51 compared to 0.38, p < 10^-16^, Mann-Whitney-Wilcoxon rank sum test).

### Microarray analysis

**Figure 4:**
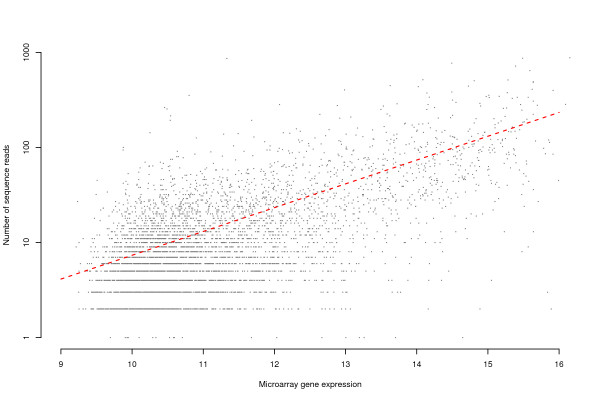
**Comparison of the gene expression measurement from the developed *Z. viviparus *microarray (x-axis, log_2 _intensity) and the number of reads produced by the massively parallel pyrosequencing (y-axis)**. The correlation was estimated to 62% (p < 10^-16^) and the red line corresponds to a Poisson regression, which explains the majority of the observed variation (adjusted R^2 ^= 52%).

A 50-mer oligonucleotide microarray was designed for large-scale gene expression analysis in *Z. viviparus*. To measure the expression of the probes and the corresponding assembled transcripts, the pool of mRNA used for the sequencing was hybridized. For 4601 transcripts with reliable annotation (see Materials), the gene expression measurement according to the probes in the annotated direction was highly correlated to the number of reads produced by the sequencing (Figure 4, correlation 62%, p < 10^-16^). Thus, the data from the gene expression microarray is consistent with the massively parallel pyrosequencing reads which validates both the assembled transcripts as well as the corresponding probe (including both a conforming binding to the correct transcript as well as a limited cross-hybridization to other transcripts). Note however, that when additional transcripts with less reliable annotation are included the correlation dropped (see Additional file 2).

### Data availability

The raw sequence data from the massively parallel pyrosequencing is available at NCBI Short Read Archive (accession number SRA007807.10), the assembled transcripts and the generated probes are available at . The data from the gene expression microarray are available as accession number GSE14957 at Gene Expression Omnibus [35].

## Discussion

We have used massively parallel pyrosequencing to characterize the transcriptome of *Zoarces viviparus*. However, sequencing and assembly of a eukaryotic transcriptome without any genomic data *a priori *is a notoriously difficult undertaking [36]. Based on 400,000 reads we assembled ~50,000 putative transcripts, a strikingly high number compared to amount of genes found in the five sequenced fish genomes (20,000–25,000). One obvious reason for the high number of transcripts is the short length of the reads, which may result in several assembled contigs and singlets for each gene. Another important factor is the process of alternative splicing, where one single gene can result in several transcripts by combining and removing different parts (usually exons). This mechanism is common in higher eukaryotes [37], including in fish [38], and the number of expressed transcripts is therefore substantially higher than the number of genes in the genome.

Another issue is the limited sequence length exhibited by massively parallel pyrosequencing. Even though the length is substantially longer than other high-throughput sequencing techniques, such as SOLiD and Illumina sequencing [29], the difference compared to Sanger-based sequencing is notable. In this study, we used a FLX Gene Sequencer, which generates 250 bases long reads, which is only a fourth of the 1000 bases long sequences which can be produced by Sanger sequencing [36]. The short sequence reads makes the assembly more difficult and thus the resulting contigs shorter. However, the average contig length (~400 bases) from our assembly is slightly longer compared to previous studies [27] and it is likely that this difference is due to the single tissue targeted in this study, but also due to the advance processing pipeline where genomic uninteresting regions, such as repeats, are removed.

For transcriptome sequencing, massively parallel pyrosequencing exhibits a high sensitivity compared to traditional Sanger-based approaches [33]. The large number of generated reads allows detection of low-abundant transcripts and the cDNA cloning step where mRNA molecules are inserted into bacterial vectors is no longer required [30]. Transcripts that previously have been hard to sequence can therefore be detected and previous studies have reported a considerable amount of reads aligning in genomic regions previously not annotated [32]. Among our assembled transcripts, ~19,000 could be aligned to at least three out of five available fish genomes and among these 3% matched regions without any prior annotation. The expression levels for these unknown transcripts were at least as high as those aligning in annotated regions and they aligned in regions with evolutionary conservation between distant species. Hence, these transcripts are likely to represent novel transcribed elements such as not yet described fish-specific genes.

Massively parallel pyrosequencing can currently only process DNA stretches of limited length. Thus, a fragmentation step is applied and as a consequence the reading direction, traditionally indicated by poly-A tail primers, is lost. Hence, the assembly pipeline therefore needs to be flexible and build contigs based on reads from both directions. Nevertheless, the direction of transcription will still be unknown for the resulting contigs. For BLAST-based sequence alignments, the knowledge of the direction of transcription is not necessary since the comparison can easily be done for both strands. However, when constructing gene expression microarrays, the direction of transcription is crucial for proper probe design, both for placing the probe on the correct strand and for evaluating probe specificity. Unfortunately, estimating the direction of transcription is intricate for short stretches of mRNA, especially for sequences without a satisfactory annotation. Even though we complemented our BLAST-based annotations with estimates from FrameFinder, an application used to identify open reading frames, a substantial part of our transcripts lacks reliable estimates of their direction of transcriptions. These transcripts may therefore be represented by probes in both directions in future gene expression assays, and thereby circumvent the problem at the cost of space on the microarray. It is finally worth to mention that the gene expression levels on our evaluation microarray are not good predictors of the direction of transcription. Only ~60% of the well-annotated transcripts have probes that had a higher expression levels in the correct direction compared to the erroneous direction (Additional file 3). This result is, however, far from surprising since the probes for the two directions will have different thermodynamic properties and specificity and therefore different intensity levels [39].

The correlation between the developed eelpout microarray and the number of reads from the massively parallel pyrosequencing was estimated to 62%. This number is strikingly high considering both the normalization of the cDNA library prior sequencing and the non-linearity typically exhibited by microarrays. Previous studies comparing hybridization- and high-throughput sequencing-based gene expression measurement techniques have reported a correlation between 46%–72% (46–62%, [40], 72–75%, [41]). In contrast to these studies, which use established previously evaluated microarrays from well-sequenced species, the eelpout microarray is designed from scratch based on less than half of the transcriptome. Thus, the correlation shows that that there is a good concordance between the developed eelpout microarray and the transcriptome sequence data, which validates both the assembled transcripts and the corresponding oligonucleotide probes.

The present study demonstrates the relative ease by which *de novo *transcriptome sequencing can be done today. The performance of the modern high-throughput sequencers is high enough that one single run is enough to cover a substantial part of the trancsriptome of a higher eukaryote. As a consequence, the assembled contigs becomes long enough to design reliable microarray probes for large-scale gene expression analysis. This development will undoubtedly spur genome-based research in many biological disciplines, especially in fields where a wide range of wild-life species are studied [42]. One prime example is ecology, where tools such as gene expression assays are needed for efficiently study the low-level mechanistic of ecosystems while other related techniques such as single nucleotide polymorphism (SNP) analysis can be used to unravel parts of the complex interplay between genetic variation and fitness in wild populations [43].

The *Zoarces viviparus *microarray developed in this study will constitute a valuable resource for marine environmental research in the Northern Europe. The platform will allow correlation of molecular-level responses, caused by exposure to various toxicants, to the unique morphological and physiological characters of *Z. viviparus*. In particular, the reproductive output in the form of size, number and quality of the embryos, can be related to gene expression changes at an individual basis. The stationary behavior of this fish species in combination with the sensitivity of gene expression biomarkers will form a unique instrument for accurate and robust monitoring of the aquatic ecosystems. The generated sequence data for the *Z. viviparus *transcriptome will also form a base to study molecular and physiological adaptations to viviparity in fish.

## Conclusion

The present study describes the first comprehensive sequencing effort of *Zoarces viviparus*, an important model organism for marine ecology research and environmental monitoring. The sequence data was generated by massively parallel pyrosequencing, a high-throughput technique with a hundred-fold higher performance than traditional capillary sequencing. The sequenced nucleotides were estimated to cover roughly 40% of the transcriptome and more than 50,000 pieces of putative transcripts were assembled. Finally, an oligonucleotide microarray were constructed and used to evaluate expression of our assembled transcripts.

## Methods

### Sampling of wild fish

*Zoarces viviparus *of both sexes were caught using fyke nets in April, June and November during the years 2005–2007 from five different locations on the Swedish west coast to cover fish with variable genetic background and seasonal changes in the expression of genes (Additional file 4). Three of the included sites are characterized by a complex pollution situation: Göteborg harbour, Stenungsund (close to petrochemical industries) and Brofjorden (close to an oil refinery). Fjällbacka, a national reference site, was together with Hönö chosen as non-polluted sites for sampling. The fish lengths were in the range of 20.8 to 27.7 cm and all fish were sexually mature. Sampling of livers was performed as describes earlier [44]. In total, livers from 18 fish were selected for cDNA synthesis.

### Exposure study

To cover otherwise rare mRNAs induced by certain toxicants, we also used livers from fish exposed in the laboratory to seven different model pollutants with different modes of action. Fish of both sexes were separated into 7 different tanks (5 fish/tank) containing 35 litres of seawater. The tanks were aerated and held at 10°C during the 72 h of exposure. The fish lengths were in the range of 20.9 to 24.8 cm and all fish were sexually mature. On day one, the fish in the three first tanks were injected intraperitoneally (0.2 ml/fish) with 15 mg paraquat/kg body weight (dissolved in 0.15 M KCl), 1 mg cadmium (CdCl_2_)/kg (dissolved in 0.15 M KCl) or 7 mg β-naphthoflavone/kg (dissolved in peanut oil). *Z. viviparus *in the following four tanks were exposed on day one and 48 h later to ethinylestradiol (EE_2_) (50 μg/l seawater, dissolved in EtOH); progesterone (100 μg/l seawater, dissolved in EtOH); methyltestosterone (100 μg/l seawater, dissolved in EtOH) or copper (CuCl_2_) (10 ng/l seawater). Tissue sampling was performed after 72 h as described earlier [44]. In total, 13 livers from the seven different exposures were selected for cDNA synthesis.

### cDNA synthesis

Total RNA was isolated by homogenization of liver samples in TRIzol (Invitrogen, Carlsbad, USA)/chloroform followed by RNeasy mini kit (Qiagen, Chatsworth, USA) for DNAse treatment and cleaning. RNA quality and quantity was analysed using Agilent 2100 Bioanalyzer (Agilent Technologies, Palo Alto, USA) and Nanodrop ND1000 (NanoDrop Technologies Wilmington, USA) respectively. Equal amount of total RNA from each of the 31 fish samples was pooled and used for cDNA synthesis. cDNA synthesis and normalization was performed by Vertis Biotechnologie AG (Freising, Germany) as follows. From the total RNA, poly(A)+ RNA was prepared. First-strand cDNA synthesis was primed with a N6 randomized primer and second-strand cDNA was synthesized according to the Gubler-Hoffman protocol. Double stranded cDNA was blunted and 454 sequencing adapter A and B were ligated to the 5' and 3' ends of the cDNA. The cDNA carrying both, adapter A and adapter B attached to its ends was selected and then amplified with PCR using a proof reading enzyme (14 cycles) according to the 454 Life Science sequencing kit (454 Life Sciences, Branford, USA). Normalization was carried out by one cycle of denaturation and reassociation of the cDNA. Reassociated ds-cDNA was separated from the remaining ss-cDNA (normalized cDNA) by passing the mixture over a hydroxylapatite column. After hydroxylapatite chromatography, the sscDNA was amplified with 8 PCR cycles.

### Massively parallel pyrosequencing

cDNA in the size range of 250 – 600 bp was eluted from a preparative agarose gel (1.5%) and the cDNA fragments were used for sequencing, which was performed at Eurofins-MWG, Germany according to [18]. Briefly, purified cDNA fragments were hybridized to DNA capture beads and each cDNA fragment individually amplified by emulsion-based clonal amplification PCR. The DNA capture beads containing amplified DNA were then deposited in individual wells of a PicoTiter plate and sequenced using the Genome Sequencer FLX instrument (454 Life Sciences, Branford, USA). Both a test run (partial plate) and a full sequence run were performed.

### Sequence pre-processing

All reads were screened for sequencing adapters located in the 5' and 3' ends. The 5' adapters were identified and removed by matching the first seven bases against TGACTAA. To prevent spurious hits for the 3' adapter, we searched the last 50 bases of all reads for TTAGTAG and when a match was found, the read was truncated right before the adapter. The dataset was then screened for redundancy in the form of duplicated reads, an artifact that is believed to be caused by multiple beads within a single PCR-reaction and/or empty wells in the fiber-optic slide [45]. After removing trailing ambiguous N's, 45,309 reads (11.3%) had an exact match to at least one other read and removing these resulted in 353,117 non-redundant sequences.

The data was then processed using SeqClean [46] using standard parameters but without any filtering based on sequence length. The genome of *Escherichia coli *[47] was used to evaluate bacterial contamination. In summary, SeqClean removed 879 reads and removed poly-A-tails from 3459 reads. To remove other unwanted regions and sequences, such as rRNA or more complex repeats, the data was applied to RepeatMasker [48,49]with RepBase Update 13.04 [50]. Default parameters were used and RepeatMasker were run in its most sensitive settings, resulting in 1,537,578 masked bases (~2%). Finally, all reads with less than 40 unambiguously nucleotides were excluded from further analysis (in total 3,136 reads removed). The resulting quality assessed sequence data thus contained ~77 million bases distributed over 349,102 reads with an average length of 221.

### Assembly and annotation

The high-quality reads were assembled using the Gene Indices cluster tool [46], which uses a multi-step procedure where the sequences first are grouped using MegaBLAST [51] and single-link hierarchical clustering which are then assembled into contigs using the CAP3 software [52]. Since massively parallel pyrosequencing is known to be less error-prone than traditional Sanger sequencing [33], we used a more stringent setting than default. In the clustering step, the overlap was set to be at least 50 bases with 95% sequence similarity and for the assembly using CAP3, the overlap percent identity cut-off was set to 95%. After assembly, all transcripts (both contigs and singlets) were annotated against the manually curated protein database UniProt [34] as well as five available fish genomes (*Gasterosteus aculeatus*, *Tetraodon nigroviridis *[53], *Takifugu rubripes *[54], *Oryzias latipes*, *Danio rerio*), the genome of *Xenopus tropicals *and the human genome [55] using WU-BLAST 2.2.6 [56,57] in blastx-mode (the number or proteins in these genomes varied and contained 356193, 29104, 23763, 48574, 25174, 35967, 28620, 61318 sequences respectively). All genomic sequence data were downloaded from Ensembl [58]. Functional annotation in the form of Gene Ontology terms [59] were also extracted from UniProt. The direction of transcription was determined both based on the best BLAST hit and the prediction from FrameFinder from the ESTate 0.50 software suite [60]. For FrameFinder, the well-described transcriptome of *Danio rerio *was used to estimate the log-odds probabilities. When applied to the full-length transcripts from the sequenced species *Gasterosteus aculeatus*, FrameFinder could correctly predict the direction of transcription for 98.8% of the genes. Note however, that these sequences are substantially longer than the assembled *Z. viviparus *transcripts. Assembled sequences with an E-value less than 10^-75 ^or a FrameFinder score higher than 100 was decided to be reliable and used for evaluation. For these sequences, BLAST and FrameFinder dissent for less than 1% of the transcripts (Additional file 5) and for those cases, the direction predicted by BLAST were chosen.

### Comparative Genomics

All contigs and singlets where aligned against all five genomes using BLAT [61] in 6-frames translational mode and a score threshold of 100 in at least three of the five species was used to define a hit. If a transcript aligned less than 500 bases from a region marked as protein coding gene, it was defined as annotated. Conservation scores based on 8-way mutiple alignments of the five fish species, *Homo sapiens*, *Mus musculus *and *Gallus gallus *were downloaded from USCS Genome Browser Database [62]. For each transcript, a conservation score per base were calculated by taking the average conservation in the alignment region and divide by the length. For comparison, a null distribution was generated by calculating analogous conservation scores for 10,000 segments at random positions, whose length and chromosome were sampled from the not annotated alignments.

### Microarray design

An oligonucleotide microarray was designed for the Geniom platform, which is fully automated system with *in situ *synthesized probes (Febit, Heidelberg, Germany). Probes for all eelpout transcripts were generated in a two-stage process. First, a preemptive clustering step, where very similar transcripts were grouped into single entities, was used to avoid losing good probes due to presumed cross-hybridization to splice variants and isogenes. Next, probes were designed using OligoArray 2.1 [63] which is an algorithm using thermodynamic approach [64] to calculate specificity and to predict secondary structures. The probe design were run using the following parameters: the probe length were set to 50 nucleotides, T_m_-range to 90°–95°, GC-content to 45%–55%, folding temperature to 70° and cross-hybridization temperature to 50°. The default settings were kept for all other parameters and no specificity check was done between transcripts within the same cluster. For sequences where no satisfactory probe could be found, a second run with a slightly less strict GC-content (45%–60%) were done. Since massively parallel pyrosequencing is dependent on a fragmentation step, where the mRNA is randomly cut into processable pieces, the strand orientation is lost and thus the direction of transcription for the generated reads and, ultimately, for the assembled contigs [18]. We therefore designed probes for all assembled transcripts and the corresponding reverse complements resulting in 39,618 (74%) and 39,733 (74%) probes respectively. In total, 117,316 probes were used and distributed over eight separate microarrays. Each array contained 15,648 spots, including controls, and were synthesized *in situ *on one single biochip using the 5' standard synthesis set for Geniom.

### Microarray Analysis

Part of the pooled hepatic RNA used for the massively parallel pyrosequencing was used to evaluate the expression of the assembled transcripts. Biotinylated cRNA was prepared using the Message Amp II-Biotin Enhanced single round Amplification kit (Ambion) starting with 1 μg of total RNA and performing the *in vitro *transcription for 14 h. Chemical fragmentation of the cRNA was performed at 94°C for 35 min, using 40 mM Tris-acetat, 100 mM K-acetate and 30 mM Mg-acetat at a concentration of 1.6 μg cRNA/μl giving an average fragment length of 100 bp. Fragmented cRNA (6 μg/array) was dissolved in hybridization buffer (final concentration: 5× SSPE, 20% formamide, 0.5 m BSA/ml, 0.1× TE, 0.1 mg mouse CotI, 0.01% Tween 20). Samples were denatured at 95°C for 3 min and hybridized to the arrays at 45°C for 15 h. Arrays were washed using 6xSSPE at 25°C and 0.5xSSPE at 45°C. Fluorescent staining of the hybridized cRNA was performed by incubating the arrays with SAPE (streptavidin, R-phycoerythrin conjugate, Invitrogen, Carlsbad, USA) diluted in 6xSSPE (5 μg SAPE/ml). Signal amplification was performed using biotinylated α-streptavidin antibodies (Vector Laboratories, Burlingame, USA) followed by and additional incubation with SAPE, according to the Consecutive Signal Enhancement protocol. Signals were detected using the CCD-based camera located in the Geniom instrument and quantified using the Geniom Wizard.

The microarray data was imported into the statistical language R 2.7.2 [65] and analyzed with Bioconductor [66]. After log_2_-transformation, differences in intensity between the eight sub-arrays were removed using median normalization [67] implemented in the R-package LIMMA [68].

## Authors' contributions

EK planned the study, performed all computational and statistical analysis, and drafted the manuscript. NA planned the study and carried out the molecular biology including the microarray assays. LF planned and supervised the study. DGJL planned and supervised the study and drafted the manuscript. All authors read and approved the final manuscript.

## Supplementary Material

Additional file 1 **A figure showing the number of homologues found in the five sequenced fish genomes, human and frog.** Click here for file

Additional file 2 **A figure showing the correlation between microarray and sequence-based gene expression data.** Click here for file

Additional file 3 **A figure showing the Differences in gene expression for all transcripts for both probes in the correct (red) and reverse (blue) direction.** Click here for file

Additional file 4 **Details regarding sampling of *Z. viviparus *along the Swedish west coast.** Click here for file

Additional file 5 **A figure showing the concordance between BLAST and FrameFinder.** Click here for file
