# Supplementary figures and images for: Characterization of the Zoarces viviparus liver transcriptome using massively parallel pyrosequencing
Source: BMC Genomics. 2009 Jul 31;10:345. doi: 10.1186/1471-2164-10-345 (PMC2725146; doi:10.1186/1471-2164-10-345)

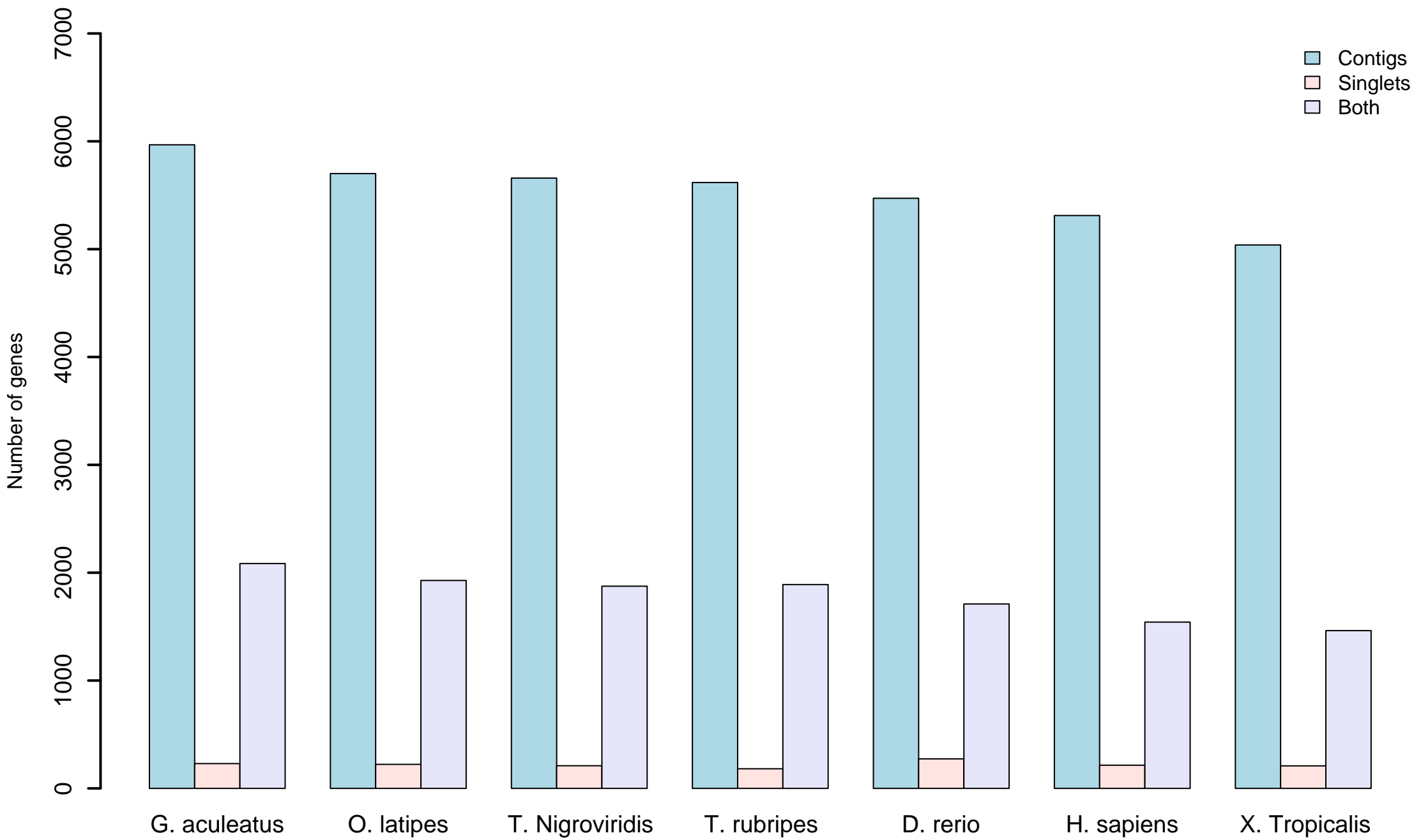

Supplement: Additional file 1 — A figure showing the number of homologues found in the five sequenced fish genomes, human and frog. [file 1471-2164-10-345-S1.pdf]

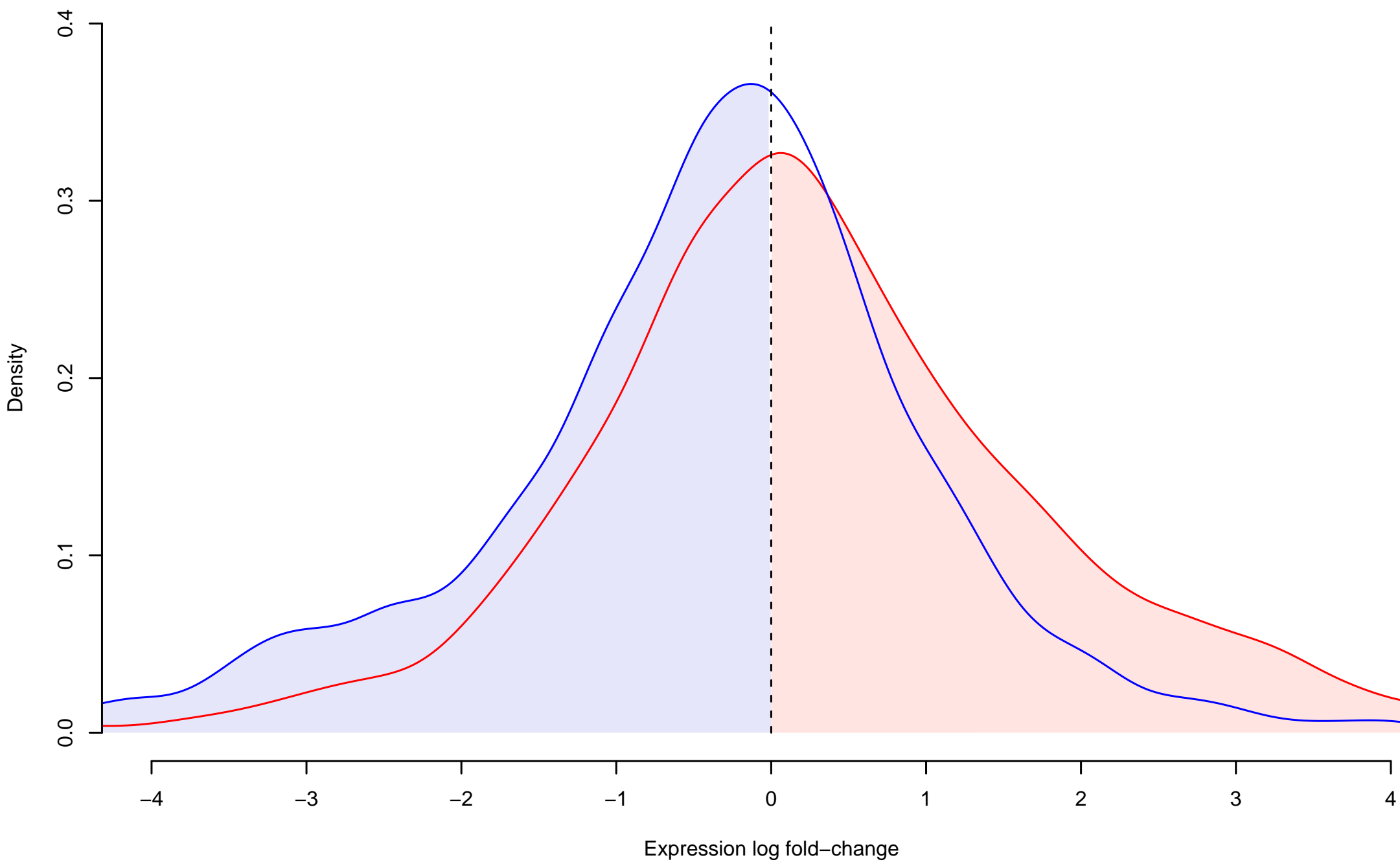

Supplement: Additional file 3 — A figure showing the Differences in gene expression for all transcripts for both probes in the correct (red) and reverse (blue) direction. [file 1471-2164-10-345-S3.pdf]

BLAST vs FrameFinder similarity

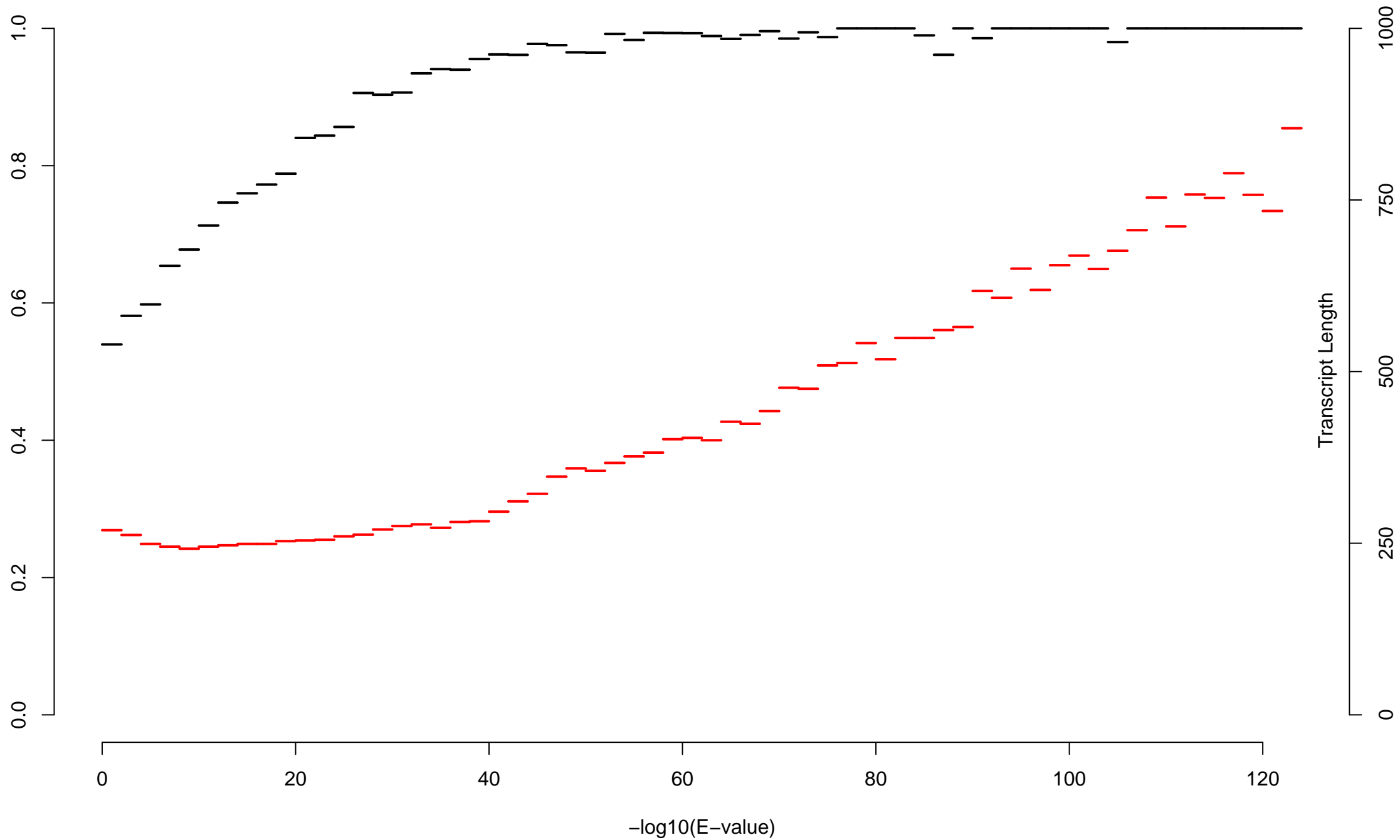

Supplement: Additional file 5 — A figure showing the concordance between BLAST and FrameFinder. [file 1471-2164-10-345-S5.pdf]
