# Supplementary material for: Characterization of the Zoarces viviparus liver transcriptome using massively parallel pyrosequencing
Source: BMC Genomics. 2009 Jul 31;10:345. doi: 10.1186/1471-2164-10-345 (PMC2725146; doi:10.1186/1471-2164-10-345)
Supplement: Additional file 2 — A figure showing the correlation between microarray and sequence-based gene expression data. [file 1471-2164-10-345-S2.pdf]

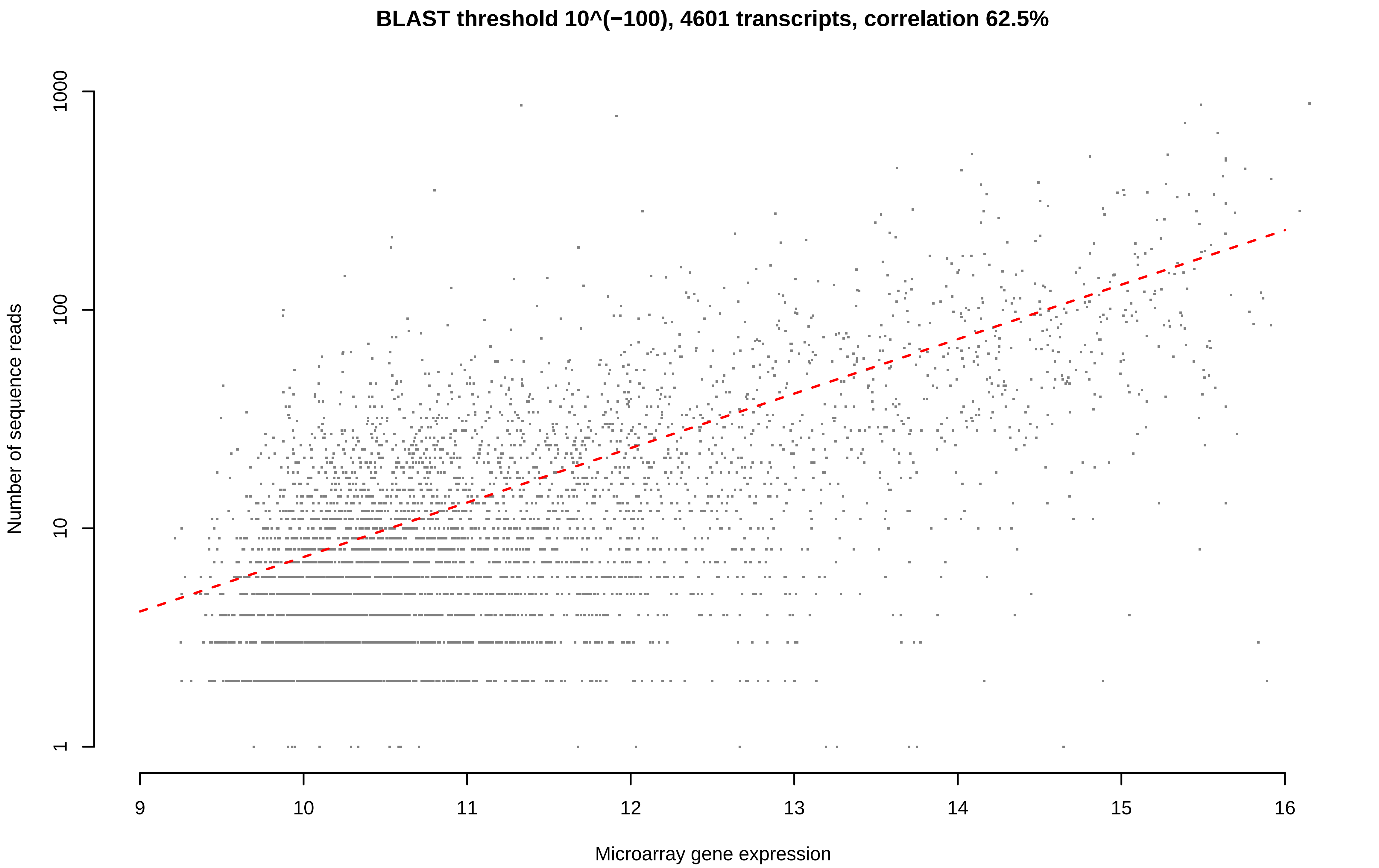

BLAST threshold 10^(−100), 4601 transcripts, correlation 62.5%
1000
100
10
1
Number of sequence reads
9
10
11
12
13
14
15
16
Microarray gene expression

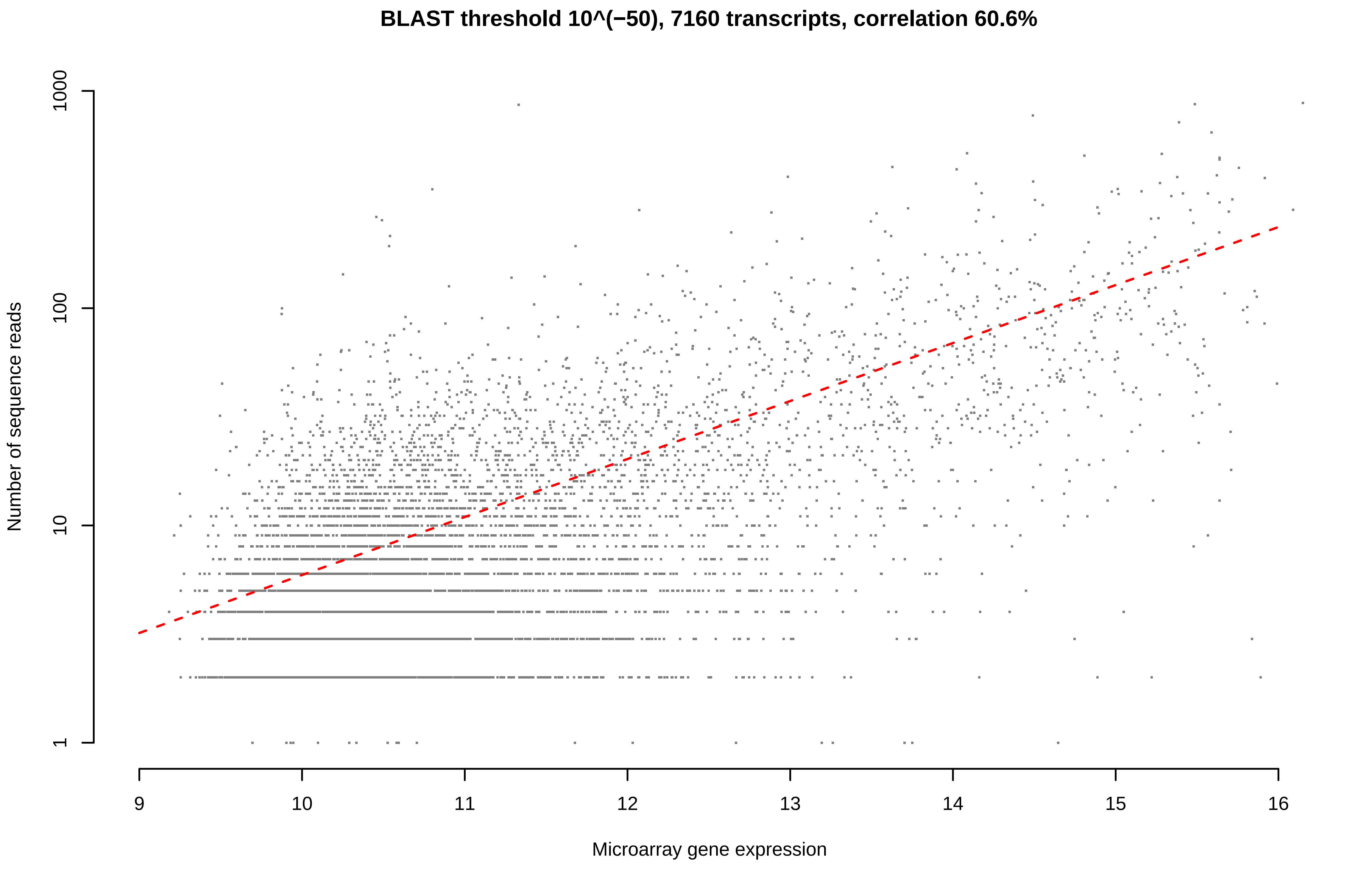

BLAST threshold 10^(−50), 7160 transcripts, correlation 60.6%
Number of sequence reads
1000
100
10
1
9
10
11
12
13
14
15
16
Microarray gene expression

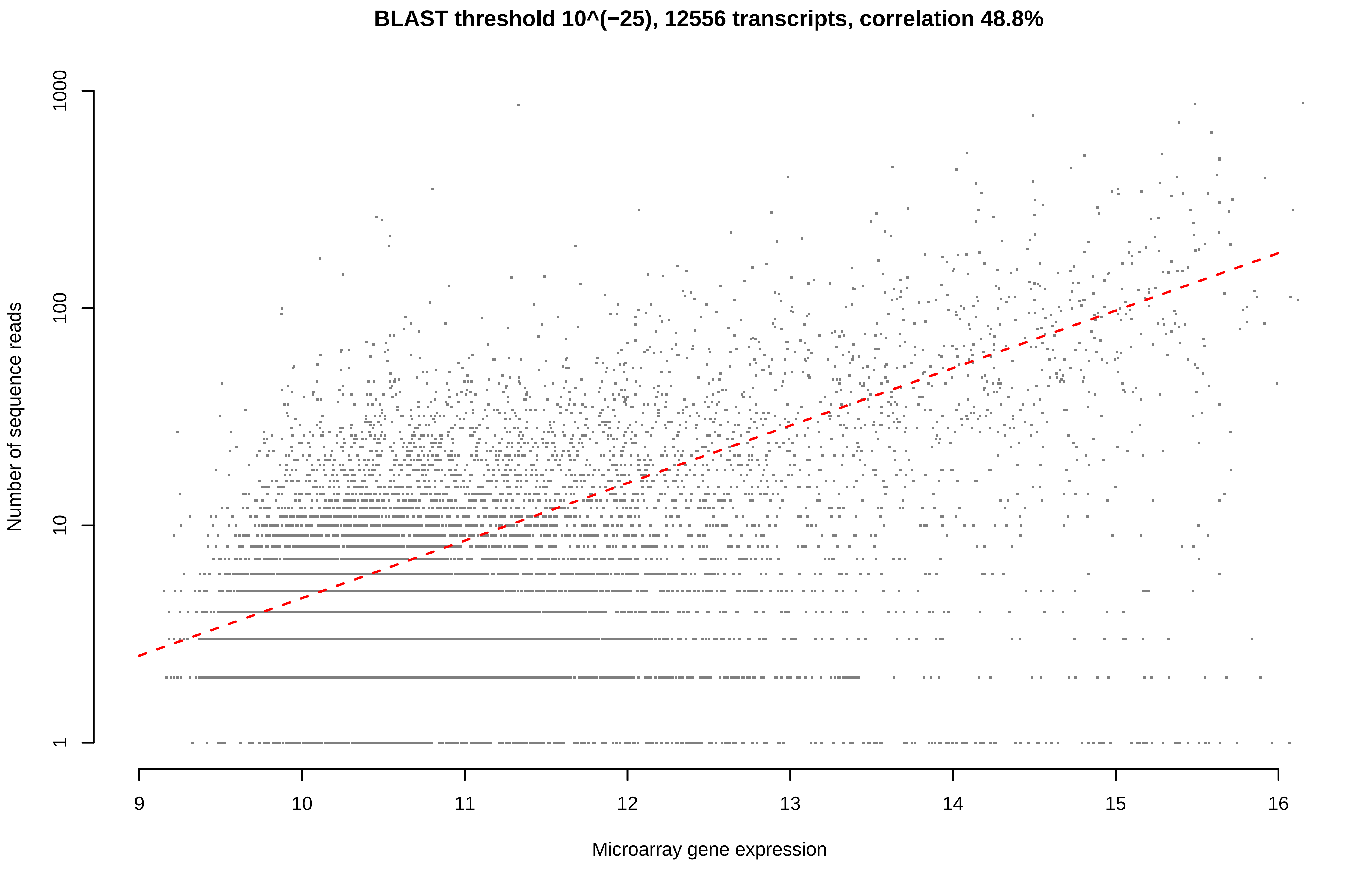
BLAST threshold 10^(−25), 12556 transcripts, correlation 48.8%
Number of sequence reads
1
10
100
1000
9
10
11
12
13
14
15
16
Microarray gene expression
